# A projection-domain iterative algorithm for metal artifact reduction by minimizing the total-variation norm and the negative-pixel energy

**DOI:** 10.1186/s42492-021-00094-w

**Published:** 2022-01-02

**Authors:** Gengsheng L. Zeng

**Affiliations:** 1 Department of Computer Science, Utah Valley University, Orem, UT 84058 USA; 2 Department of Radiology and Imaging Sciences, University of Utah, Salt Lake City, UT 84108 USA

**Keywords:** Analytical image reconstruction, Metal artifact reduction, Projection-domain iterative algorithm, X-ray computed tomography

## Abstract

Metal objects in X-ray computed tomography can cause severe artifacts. The state-of-the-art metal artifact reduction methods are in the sinogram inpainting category and are iterative methods. This paper proposes a projection-domain algorithm to reduce the metal artifacts. In this algorithm, the unknowns are the metal-affected projections, while the objective function is set up in the image domain. The data fidelity term is not utilized in the objective function. The objective function of the proposed algorithm consists of two terms: the total variation of the metal-removed image and the energy of the negative-valued pixels in the image. After the metal-affected projections are modified, the final image is reconstructed via the filtered backprojection algorithm. The feasibility of the proposed algorithm has been verified by real experimental data.

## Introduction

The metal artifacts in X-ray computed tomography (CT) are mainly due to the beam hardening effects [1–4]. The linear attenuation coefficient in a material is a function of the X-ray energy. X-ray CT systems normally use polychromatic beams, and the various energy components are not attenuated uniformly. The lower energy component of X-ray spectrum is more easily to be attenuated or even completely adsorbed when traveling through metals. The beam hardening effects make the sinogram values deviate from the assumption that the sinogram values are the line-integrals of the attenuation coefficients in the object.

Many techniques are commonly used to reduce the metal beam hardening effects. In clinical scans, the metal materials and the X-ray source settings are known. Model-based iterative algorithms [5–12] and iterative segmentation-based interpolation methods [13–20] can be used by taking the advantage of the available knowledge of the metals, the soft tissues, the bones, and X-ray source settings.

In this paper, we consider a more challenging situation where the objects of interest, the metal materials and the X-ray source settings are all unknown. Thus, model-based iterative reconstruction methods are not effective here.

There are other methods to be considered. Bayesian algorithms, for example, the total variation (TV) norm minimization can be considered [21–25]. The metal artifacts appear as bright or dark streaks, radiated from the metals. The bright parts are the positive overshoots; the dark parts are the negative undershoots. The negative undershoots may result in negative image pixels. Our proposed algorithm is to minimize the TV of the metal-removed image [25] and the energy of the negative image pixels [26]. The algorithm variables are the metal affected sinogram values.

We must point out that it is not straightforward to reflect the negative image pixel constraint in the sinogram domain. It is not equivalent to setting the associated sinogram values to zero or positive [26].

## Methods

This paper is inspired by the two observations that the metal artifacts often result in dark/bright streaks radiating from the metals [25] and negative image pixels around the metals [26]. The streaking artifacts increase the TV norm of the non-metal regions of the image. The attenuation coefficients cannot be negative. These two effects were dealt with in two papers, separately [16, 26]. The goal of this current paper is to combine these two methods into one.

Let the measured sinogram (i.e., line integrals) be *P*, which can be divided into two parts: the metal affected part *P*_*M*_ and the metal unaffected part *P*_*notM*_. This separation can be readily achieved by performing a raw filtered backprojection (FBP) reconstruction, thresholding, and forward projection. The objective function is hence

1 $$ {\displaystyle \begin{array}{c}\mathrm{Objectivefunction}=T(P)={\beta}_1{T}_1+{\beta}_2{T}_2\\ {}={\beta}_1{\left\Vert G\left\{F(P)\right\}\right\Vert}_{TV}+{\beta}_2{\left\Vert \min \Big\{0,F(P)\Big\}\right\Vert}^2\end{array}} $$

where the first term is the TV norm, and the second term is the squared *L*_2_ norm. In Formula (1), *F* denotes the FBP reconstruction operator, *G* is the operator that removes the metals from the image, and *β*_1_ and *β*_2_ are the parameters balancing the two norms. In the gradient descent algorithm, *β*_1_ and *β*_2_ also act as the step sizes (also, known as the relaxation factors).

We minimize this objective function with respect to unknowns *P*_*M*_ with a gradient descent algorithm. It is noticed that neither of the two terms in Formula (1) is differentiable. Sub-gradients are used to combat this difficulty [27].

A sub-gradient is a ‘surrogate’ of the gradient: it coincides with the gradient whenever a gradient exists, and it generalizes the notion of gradient at points where the function is non-differentiable. Let us consider a point of interest and the function is non-differentiable at this point. A sub-gradient is evaluated as follows. We find the left-gradient by taking the limit from the left and the right-gradient by taking the limit from the right, which are also known as the directional gradients. Any values between these two left and right values can be used as the sub-gradient.

We now use the absolute function |*x*| to illustrate how to find the sub-gradient. When *x* > 0, the sub-gradient is the same as the gradient: 1. When *x* < 0, the sub-gradient is the same as the gradient: −1. When *x* = 0, the function |*x*| is non-differentiable. The left-gradient is −1 and the right-gradient is 1. The sub-gradient at *x* = 0 can be any value in the interval [− 1, 1].

In our implementation, three values of *β*_1_ were tested: 0, 0.002 and 0.004; two values of *β*_2_ were tested: 0 and 5.

### The FBP algorithm

In Formula (1), *F*(*P*) is a linear algorithm that can be deposed into two steps. The first step is to convolve the sinogram *P* along the detector dimension with a convolution kernel *h*(*n*):

2 $$ h(n)=\left\{\begin{array}{ccc}\frac{1}{4}& if& n=0,\\ {}-\frac{1}{{\left( n\pi \right)}^2}& if& n\  is\  odd,\\ {}0& otherwise.& \end{array}\right. $$

The second step is the backprojection. Let the FBP reconstruction from *P* be *X*. If both *P* and X are represented in the vector forms, *F*(*P*) can be written as matrix multiplication

3 $$ X= FP $$

where *F* is a matrix.

For a given threshold value *t*, a metal image is obtained by modifying the image *X*. If a pixel in *X* is greater than *t*, its value is set to 1, otherwise its value is set to 0. The forward projection of *X* generates a ‘shadow’ in the projection domain. If the shadow values are positive, the corresponding sinogram values are called metal-affected projections, *P*_*M*_. If the shadow values are zero, the corresponding sinogram values are called metal-not-affected projections, *P*_*notM*_. The sinogram *P* is thus divided into two parts: *P*_*M*_ and *P*_*notM*_. The projection values in *P*_*M*_ are treated as variables in the proposed algorithm.

In Formula (1), the metal removed FBP image, *G*{*F*(*P*)} = *G*{*X*}, is almost the same as *X*, except that if a pixel value is greater than *t*, its value is set to 0.

In Formula (1), min{0, *F*(*P*)} = min {0, *X*} is almost the same as *X*, except that if a pixel value is positive, its value is set to 0. Here, the ‘min’ function is an element-wise function.

### Optimization of the objective function (Formula 1) by the gradient descent algorithm

A gradient descent algorithm to minimize the objective function *T*(*P*) in Formula (1) can be expressed as

4 $$ {P}_M^{\left(k+1\right)}={P}_M^{(k)}-D\left[{\beta}_1\nabla {T}_1(P)+{\beta}_2\nabla {T}_2(P)\right] $$

where the super script (*k*) is the iteration index. The projection vector *P* consists of two parts: the metal affected part *P*_*M*_ and the metal not-affected part *P*_*notM*_. The metal not-affected part *P*_*notM*_ does not get updated from iteration to iteration. The matrix *D* in Formula (4) is a diagonal matrix with 0’s and 1’s, which discards the entries in *P*_*notM*_. The parameter *β*_1_ and *β*_2_ in Formula (4) controls the step sizes of the gradient descent algorithm for the two different norms, respectively. In Formula (4), ∇*T*_1_ is the gradient of the TV norm of the image with the metals removed, and ∇*T*_2_ is the gradient of the energy of the negative pixels in the image. These two gradients are calculated with respect to the projections *P*. These two gradients are discussed further in the following two sub sections.

### Minimization of the first term in the objective function (Formula 1)

Let *Y* = *G*{*F*(*P*)} = *G*{*X*} be the metal-removed FBP reconstruction. The image *Y* is represented in a two-dimensional array and *y*_*i,j*_ is its pixel value at the *i*th row and *j*th column. The TV norm of *Y* is defined as

5 $$ {T}_1={\sum}_{i,j}\sqrt{{\left({y}_{i,j}-{y}_{i,j+1}\right)}^2+{\left({y}_{i,j}-{y}_{i+1,j}\right)}^2} $$

The partial derivative of *T*_1_ with respect to pixel (*i, j*) (if exists) is readily calculated as

6 $$ {\displaystyle \begin{array}{c}{u}_{i,j}=\frac{\partial {T}_1}{\partial {y}_{i,j}}\\ {}\begin{array}{c}=\frac{\left({y}_{i,j}-{y}_{i,j+1}\right)+\left({y}_{i,j}-{y}_{i+1,j}\right)}{\sqrt{{\left({y}_{i,j}-{y}_{i,j+1}\right)}^2+{\left({y}_{i,j}-{y}_{i+1,j}\right)}^2}}\\ {}\begin{array}{c}+\frac{y_{i,j}-{y}_{i,j-1}}{\sqrt{{\left({y}_{i,j-1}-{y}_{i,j}\right)}^2+{\left({y}_{i,j-1}-{y}_{i+1,j-1}\right)}^2}}\\ {}+\frac{y_{i,j}-{y}_{i-1,j}}{\sqrt{{\left({y}_{i-1,j}-{y}_{i-1,j+1}\right)}^2+{\left({y}_{i-1,j}-{y}_{i,j}\right)}^2}}\end{array}\end{array}\end{array}} $$

When the quantity under a square root is zero, the gradient *u*_*i,j*_ is not well-defined. In this case, we use the sub-gradient and assume the sub-gradient *u*_*i,j*_ to be zero

7 $$ {u}_{i,j}=0 $$

The gradient of *T*_1_ given in Formula (6) is with respect to the image pixels *y*_*i,j*_. However, the gradient decent algorithm (Formula 4) requires the gradient of *T*_1_ be with respect to the projections *P*.

Realizing that *Y* is in the image domain, the algorithm variables are in the projection domain related by the mapping

8 $$ P= AY $$

where, *A* represents the forward projection matrix. The matrix *A* maps the image into its projections. This same matrix *A* can map the gradient *U* into the projection domain as *AU*, where the entries of *U* are *u*_*i,j*_. We have

9 $$ \nabla {T}_1(P)= AU $$

### Minimization of the second term in the objective function (Formula 1)

Let *Z* = min {0, *F*(*P*)} = min {0, *X*} be the negative pixels in the FBP reconstruction *X*. The energy of the negative image pixels is the square of the L_2_ norm of *Z* given as

10 $$ {T}_2={\left\Vert Z\right\Vert}_2^2={\left\Vert \min \left\{0, FP\right\}\right\Vert}_2^2 $$

To find the gradient ∇*T*_2_(*P*) , that is, *∂T*_2_/*∂p*_*j*_, is not straightforward, because the ‘min’ function in Formula (10) makes *T*_2_ non-differentiable. We can use the subdifferential concept to find the gradient of *∂T*_2_/*∂p*_*j*_ as [27]

11 $$ \nabla {T}_2=2{F}^T\min \left\{0, FP\right\}=2{F}^TZ $$

where *F*^*T*^ represents forward projection followed by ramp filtering. The operator *F*^*T*^ maps an image into the projection domain.

### Proposed gradient descent algorithm to estimate *P*_*M*_

Combining the gradients above, the proposed gradient descent iterative algorithm (Formula 4) to estimate *P*_*M*_ can given as

12 $$ {P}_M^{\left(k+1\right)}={P}_M^{(k)}-D\left[{\beta}_1\tanh (AU)+{\beta}_2\ {F}^TZ\right] $$

where tanh(∇*T*_1_) is used in place of ∇*T*_1_ in Formula (4). The purpose of the hyperbolic tangent function ‘tanh’ is to hard limit the TV gradient values, making the iterative algorithm more stable.

The proposed algorithm is different from the commonly used iterative algorithms in the sense that the sinogram values in a common iterative reconstruction algorithm are never altered. Also, the proposed algorithm does not have a data fidelity term in the objective function (Formula 1). Our final image is obtained by the FBP algorithm, after *P*_*M*_ is modified. The initial value of the metal affected part *P*_*M*_ is the measured value.

We tested the proposed algorithm with some unknown airport bags, which contained some unknown metals. The projection sinograms were provided by the US Department of Homeland Security. The data sets were acquired with unknown kVp and unknown current settings. The original sinograms were converted into parallel-beam geometry with 180 views over 180° and with 597 detection channels (i.e., detector bins).

## Results

**Fig. 1:**
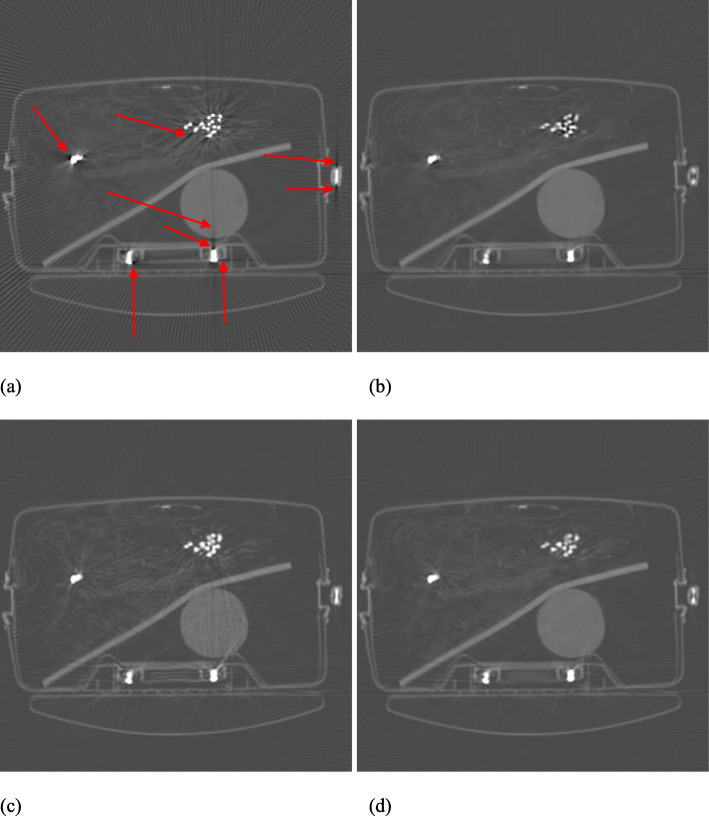
Reconstruction of bag #1. **a** Raw FBP; **b** TV-term only; **c** Non-negativity-term only; **d** Combined. Some artifacts are marked by the red arrows

**Fig. 2:**
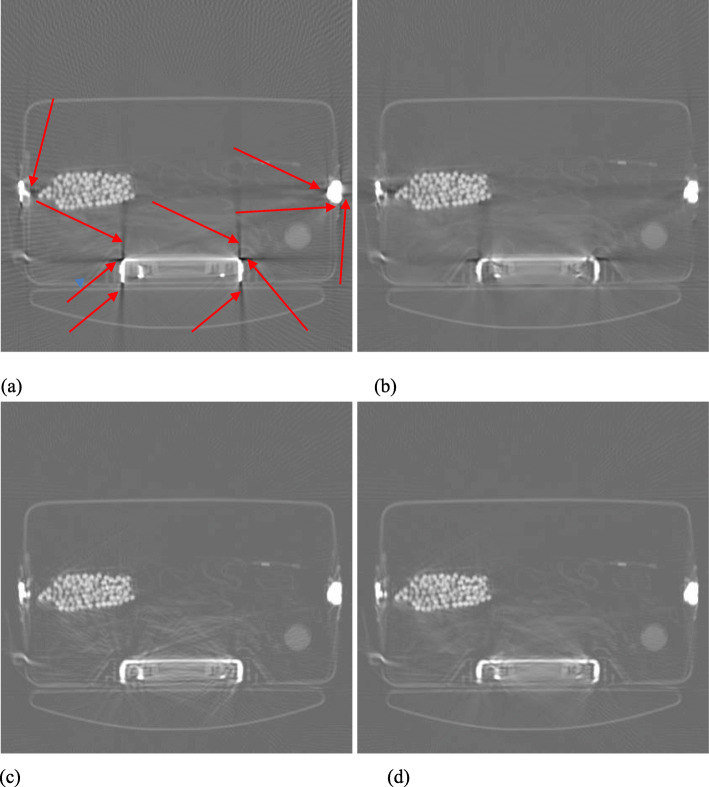
Reconstruction of bag #2. **a** Raw FBP; **b** TV-term only; **c** Non-negativity-term only; **d** Combined. Some artifacts are marked by the red arrows

**Fig. 3:**
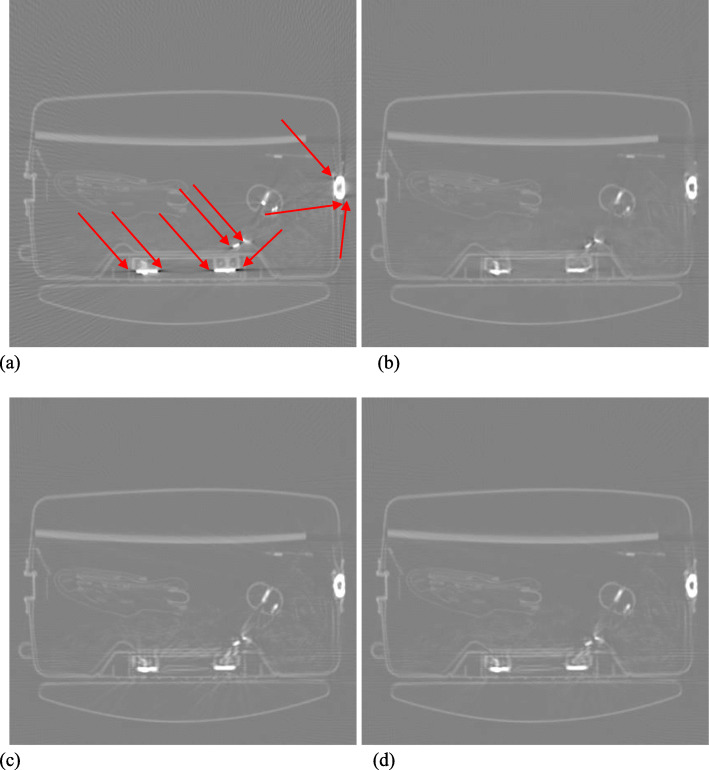
Reconstruction of bag #3. **a** Raw FBP; **b** TV-term only; **c** Non-negativity-term only; **d** Combined. Some artifacts are marked by the red arrows

**Fig. 4:**
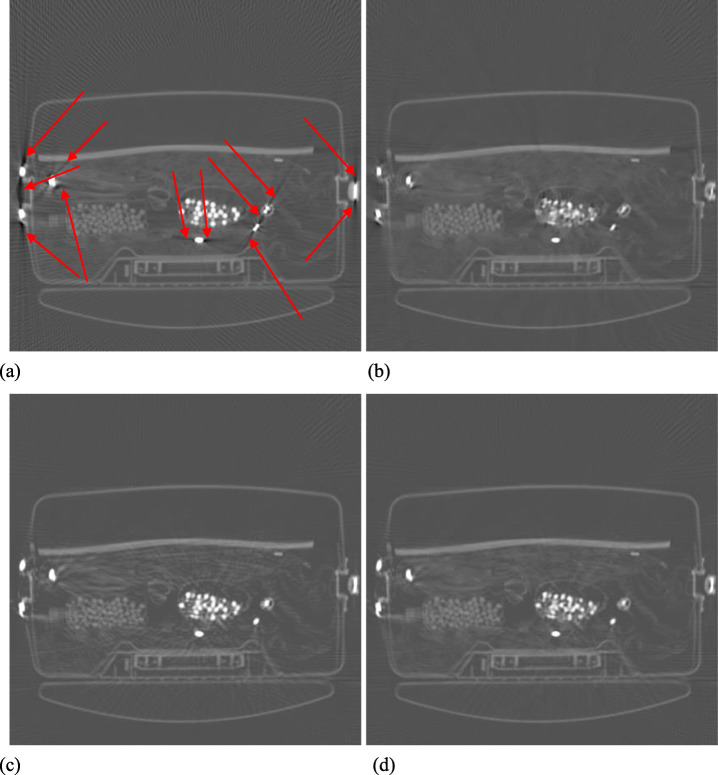
Reconstruction of bag #4. **a** Raw FBP; **b** TV-term only; **c** Non-negativity-term only; **d** Combined. Some artifacts are marked by the red arrows

**Fig. 5:**
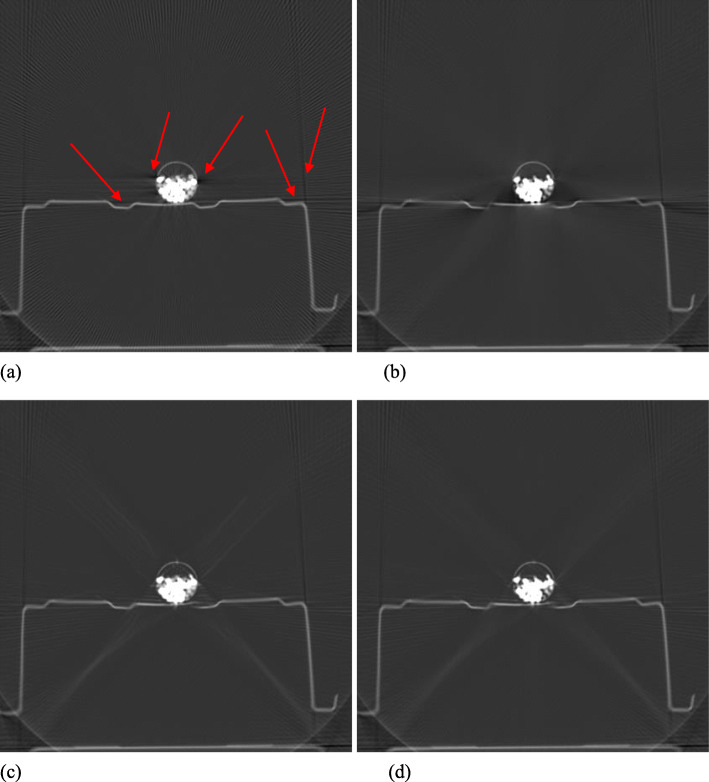
Reconstruction of bag #5. **a** Raw FBP; **b** TV-term only; **c** Non-negativity-term only; **d** Combined. Some artifacts are marked by the red arrows

Figures 1, 2, 3, 4 and 5 show the reconstruction results of 5 different unknown airport bags. Each figure consists of 4 images: the original raw FBP reconstruction; the TV-norm-only (with *β*_1_ = 0.004 and *β*_2_ = 0) reconstruction; the negativity-pixel-energy-only (with *β*_1_ = 0 and *β*_2_ = 5); the combined reconstruction (with *β*_1_ = 0.004 and *β*_2_ = 5). All images have the same display window. Black color represents negative values and white color represents large positive values. In order to visualize the metal artifacts, the display window is determined by the raw FBP reconstruction using the original measured sinogram. The display window is airport bag specific. The 1/3 of the maximum raw FBP image value is mapped to the gray level 255 (white). Any value larger is set to 255. The metal pixels are much brighter than other pixels in the image. If we do not clip the very bright metal pixels, we cannot see other structures in the image. The minimum raw FBP value (which is negative) is mapped to the gray level 0 (black). The mapping is linear in the range below 1/3 of the maximum value. All 4 images in the figure use the same gray-scale mapping. Therefore, back pixels are negative.

The number of iterations for all studies in this paper was 400 in the gradient descent algorithm. The image array size was 420 × 420, and the pixel size was 0.92 mm. The proposed algorithm was implemented using MATLAB®, and the computational time for 400 iterations was 145 s.

Because the ground truth is unknown to us, it is inappropriate to perform quantitative evaluations. In this paper, we only make human visual inspections on the reconstructions, focusing on the severeness of the artifacts. Two evaluation metrics were used. One metric was the TV norm of the reconstructed image with metals removed. The other metric was the energy of the negative pixels in the reconstructed image.

It is seen that all raw FBP images (shown at the upper-left conner) contain severe black streaking artifacts, indicating streaks of negative values. When the objective function only has the TV-norm term, that is, *β*_2_ = 0, most black streaks are removed from the images (shown at the upper-right conner); unfortunately, many low-contrast structures are lost at the same time. The lower-left images are obtained using only the negative-pixel-energy minimization (i.e., *β*_1_ = 0), and the black streaking artifacts are effectively removed from the resulting reconstructions. Compared with the upper-right images, the lower-left images look noisier and may contain some new bright streaking artifacts. If both of the two terms are used in the objective function (Formula 1), the resultant images are shown at the lower-right conner, which enjoy the benefits from both terms in the objective function (Formula 1).

**Fig. 6:**
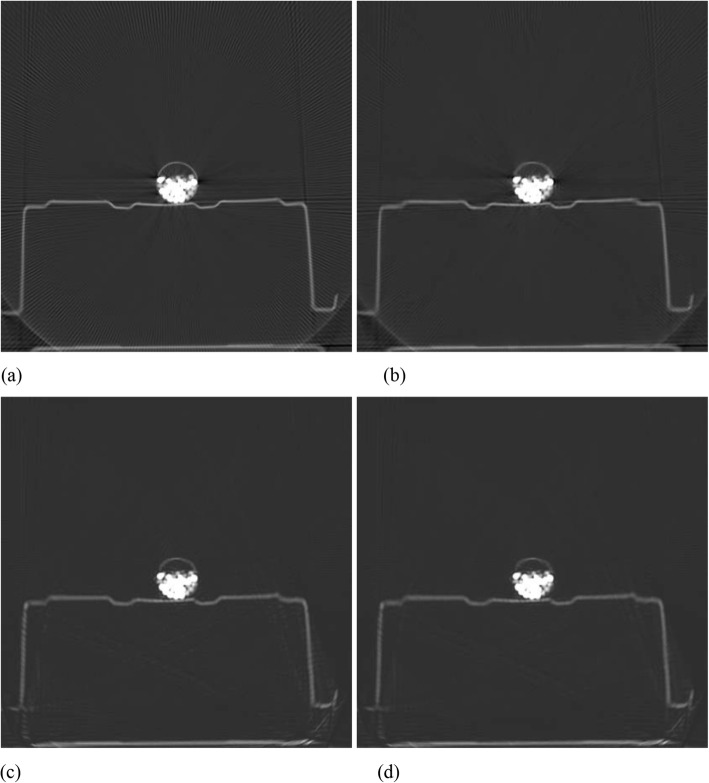
Reconstruction of bag #5 (New threshold value to segment the metal image and new *β*_1_ = 0.0002). **a** Raw FBP; **b** TV-term only; **c** Non-negativity-term only; **d** Combined

**Fig. 7:**
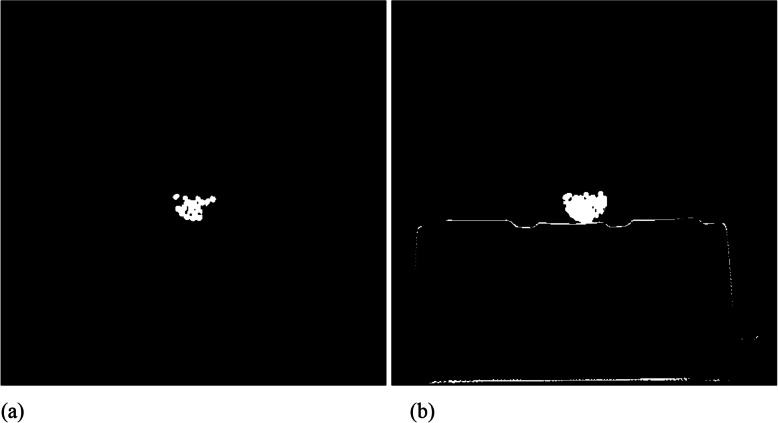
Metal images for bag #5. **a** The segmentation threshold *t* = 1/3 of the maximum raw FBP image pixel value; **b** The segmentation threshold *t* = 1/10 of the maximum raw FBP image pixel value

In Fig. 5, the metal reduction methods fail to remove the dark streaking artifacts. On the other hand, Fig. 6 shows better results for the same airport bag. The different results in these two figures are mainly due to the different setup in metal image segmentation. In Figs. 1, 2, 3, 4 and 5, the FBP image pixels are segmented into the metal image if the image values are greater than *t* = 1/3 of the maximum image value. However, in Fig. 6, the FBP image pixels are segmented into the metal image if the image values are greater than 1/10 of the maximum image value. The change of the segmentation threshold results in different metal maps, as illustrated in Fig. 7. After changing the threshold value *t* from 1/3 to 1/10, we must change *β*_1_ = 0.004 to a much smaller value *β*_1_ = 0.002, otherwise the resultant image is too smooth and lots of image details disappear.

Tables 1, 2, 3, 4, 5 and 6 list the maximum and minimum values for each image in Figs. 1, 2, 3, 4, 5 and 6, respectively. It is noticed that the images generated from the processed sinograms still have negative image pixels. The residual negative pixels are most likely due to the angular aliasing artifacts because we use only 180 views in our sinogram data. A typical X-ray CT scan has approximately 1000 views. The tables also list the evaluation metrics: the TV norm of the reconstructed image with metals removed and the energy of the negative pixels in the reconstructed image.

**Table 1:** Values of maximum, minimum, metal-free region TV, and NPE in reconstructions of bag #1

Iterations	*β*_1_	*β*_2_	Min	Max	TV	NPE
0, raw FBP	NA	NA	−0.1591	1.2781	1501.4	3.1231
1000	0.004	0	−0.1365	1.0643	835.6338	0.3426
1000	0	5	−0.0133	1.1837	1014.6	0.0631
1000	0.004	5	−0.0134	1.1408	869.5945	0.0712

**Table 2:** Values of maximum, minimum, metal-free region TV, and NPE in reconstructions of bag #2

Iterations	*β*_1_	*β*_2_	Min	Max	TV	NPE
0, raw FBP	NA	NA	−0.3000	1.3420	1148.4	8.3468
1000	0.004	0	−0.11825	1.1570	834.2642	3.1681
1000	0	5	−0.0196	1.3585	1150.3	0.1340
1000	0.004	5	−0.0206	1.3130	906.2427	0.1476

**Table 3:** Values of maximum, minimum, metal-free region TV, and NPE in reconstructions of bag #3

Iterations	*β*_1_	*β*_2_	Min	Max	TV	NPE
0, raw FBP	NA	NA	−0.3776	1.2154	1144.3	4.7415
1000	0.004	0	−0.0843	1.0329	891.2476	1.6540
1000	0	5	−0.0191	1.1270	1025.8	0.3326
1000	0.004	5	−0.0223	1.0872	964.3871	0.3294

**Table 4:** Values of maximum, minimum, metal-free region TV, and NPE in reconstructions of bag #4

Iterations	*β*_1_	*β*_2_	Min	Max	TV	NPE
0, raw FBP	NA	NA	−0.1868	1.3204	1379.1	1.9616
1000	0.004	0	−0.0757	1.2775	1006.9	0.4434
1000	0	5	−0.0155	1.3992	1226.9	0.1255
1000	0.004	5	−0.0164	1.4060	1062.4	0.1379

**Table 5:** Values of maximum, minimum, metal-free region TV, and NPE in reconstructions of bag #5

Iterations	*β*_1_	*β*_2_	Min	Max	TV	NPE
0, raw FBP	NA	NA	−0.0347	0.4676	472.6624	0.3366
1000	0.004	0	−0.0371	0.5137	220.4515	0.0558
1000	0	5	−0.0340	0.5487	225.6903	0.0201
1000	0.004	5	−0.0344	0.5041	220.1787	0.0222

**Table 6:** Values of maximum, minimum, metal-free region TV, and NPE in reconstructions of bag #5 (New threshold value to segment the metal image and new *β*_1_ = 0.0002)

Iterations	*β*_1_	*β*_2_	Min	Max	TV	NPE
0, raw FBP	NA	NA	−0.0347	0.4676	472.6624	0.3366
1000	0.0002	0	−0.0342	0.5251	165.2475	0.0190
1000	0	5	−0.0030	0.5243	180.0589	0.00015141
1000	0.0002	5	−0.0031	0.5252	144.7916	0.0011

**Fig. 8:**
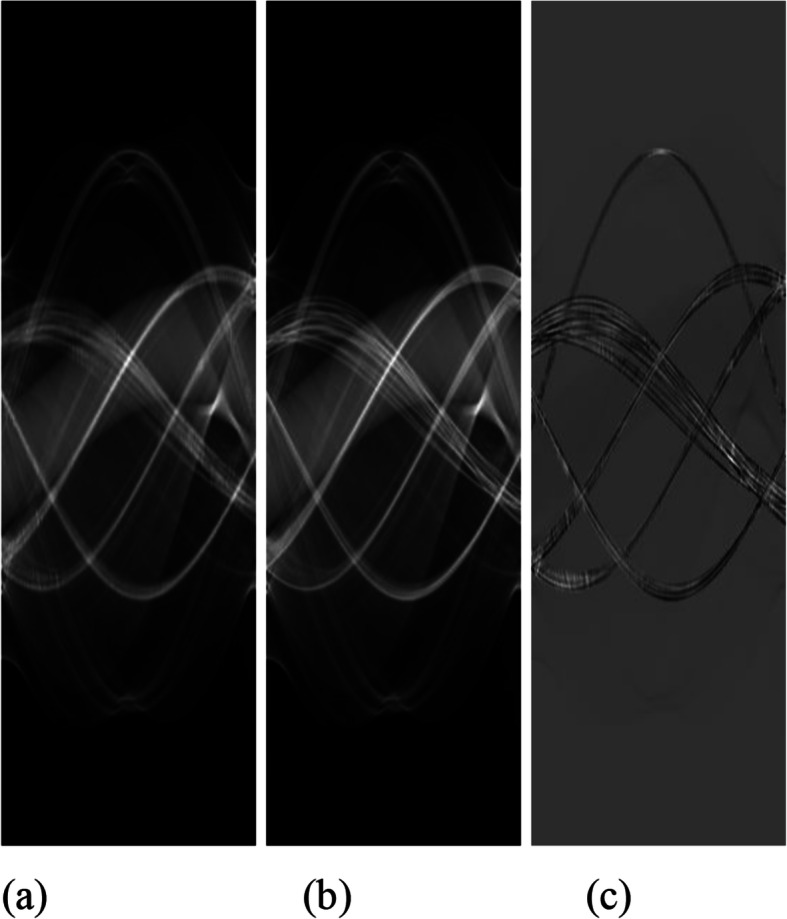
Sinograms of bag #1. **a** Processed with *β*_1_ = 0.004 and *β*_2_ = 5; **b** Raw; **c** Difference

Figure 8 compares bag #1 sinograms before and after processing. Figure 8(a) is for the case of *β*_1_ = 0.004 and *β*_2_ = 5. Figure 8(b) represents the raw measurements. The difference image [(a) – (b)] is shown at Fig. 8(c). It is observed that most sinogram values are not altered by the iterative algorithm.

## Discussion

This paper combines two methods into one. These two methods use two different objective functions. They try to reduce the metal artifacts by using two different strategies. One method tries to reduce the artifacts that have oscillations; the other method tries to reduce the artifacts that have negative overshoots. By combining these two methods, we have a better chance to reduce metal artifacts that have both oscillating and negative overshoots features. One potential limitation is that those two methods may work in the opposite ways, making none of the methods effective. Another potential limitation is that the combined method has some hyper parameters β1 and β2 to be adjusted. There are no general rules to set them.

The ratio of these two parameters determines which objective function should be more dominating. Their values also affect the convergence rate of the gradient descent algorithm. Usually, larger values of *β*_1_ and *β*_2_ result in fast convergence. If their values are too large, the gradient descent algorithm may diverge. There is no clear relationship between the beta values and the threshold value. The values of *β*_1_ and *β*_2_ should be set for every new data set and every new object. The value of *β*_2_ may not be sensitive to the data set or object. There are no general rules how to set these two hyper parameters. It is observed from Tables 1, 2, 3, 4, 5 to 6 that the TV norm values *T*_1_ are much larger than the energy of negative pixel energy (NPE) values *T*_2_. Therefor, *β*_1_ is much smaller than *β*_2_.

The setting of the hyper parameters is data dependent. One setting works well for one object but does not work well for another object. This newer value of *β*_1_ for bag #5 did not work well for other data sets. The hyper parameters are set by trial-and-error for each case.

From our experimental results, there is no significant difference between the results of non-negativity-term only and combined. The minimum and maximum values of the two results are also very close. This observation implies that among the two methods, the method to reduce the negative overshoot is more effective than the TV minimization method for the cases presented.

## Conclusions

This paper proposes a projection-domain iterative algorithm to estimate metal affected projections using an image-domain objective function. The objective function does not have a data fidelity term. It consists of the TV norm and the energy of the negative pixels. Real CT scans with unknown objects and unknown metals are used to test the feasibility of the proposed algorithm. The metals cause severe streaking artifacts in the raw FBP reconstructions. Those streaking artifacts are successfully removed by the proposed algorithm.

Results of two special cases, *β*_1_ = 0 and *β*_2_ = 0, are also compared. When *β*_2_ = 0, the algorithm only minimizes the image TV norm. The images with TV norm minimization tend to be oversmoothed, and some low contrast structures may get lost. When *β*_1_ = 0, the algorithm only minimizes the image NPE. The images with NPE minimization contain more low contrast structures but may be noisy. When both terms are used in the objective function (1), the final images are more balanced between details and noise control. In all three cases discussed above, the metal streaking artifacts are effectively reduced.

## Data Availability

Not applicable.

## Abbreviations

CT: Computed tomography
FBP: Filtered backprojection
NPE: Negative pixel energy
TV: Total variation
